# An integrated health sector response to violence against women in Malaysia: lessons for supporting scale up

**DOI:** 10.1186/1471-2458-12-548

**Published:** 2012-07-24

**Authors:** Manuela Colombini, Susannah H Mayhew, Siti Hawa Ali, Rashidah Shuib, Charlotte Watts

**Affiliations:** 1 Department of Global Health and Development, London School of Hygiene and Tropical Medicine, London, UK; 2 School of Health Sciences, Universiti Sains Malaysia, Gelugor, Penang, Malaysia; 3 Women's Development Research Centre (KANITA), Universiti Sains Malaysia, Gelugor, Penang, Malaysia

**Keywords:** Gender-based violence, Scale up, Integration, Intimate partner violence, Malaysia

## Abstract

**Background:**

Malaysia has been at the forefront of the development and scale up of One-Stop Crisis Centres (OSCC) - an integrated health sector model that provides comprehensive care to women and children experiencing physical, emotional and sexual abuse. This study explored the strengths and challenges faced during the scaling up of the OSCC model to two States in Malaysia in order to identify lessons for supporting successful scale-up.

**Methods:**

In-depth interviews were conducted with health care providers, policy makers and key informants in 7 hospital facilities. This was complemented by a document analysis of hospital records and protocols. Data were coded and analysed using NVivo 7.

**Results:**

The implementation of the OSCC model differed between hospital settings, with practise being influenced by organisational systems and constraints. Health providers generally tried to offer care to abused women, but they are not fully supported within their facility due to lack of training, time constraints, limited allocated budget, or lack of referral system to external support services. Non-specialised hospitals in both States struggled with a scarcity of specialised staff and limited referral options for abused women. Despite these challenges, even in more resource-constrained settings staff who took the initiative found it was possible to adapt to provide some level of OSCC services, such as referring women to local NGOs or community support groups, or training nurses to offer basic counselling.

**Conclusions:**

The national implementation of OSCC provides a potentially important source of support for women experiencing violence. Our findings confirm that pilot interventions for health sector responses to gender based violence can be scaled up only when there is a sound health infrastructure in place – in other words a supportive health system. Furthermore, the successful replication of the OSCC model in other similar settings requires that the model – and the system supporting it – needs to be flexible enough to allow adaptation of the service model to different types of facilities and levels of care, and to available resources and thus better support providers committed to delivering care to abused women.

## Background

There is growing recognition of the substantial health impacts of violence against women, including numerous physical, reproductive health and mental health consequences, both short and long-term [1,2]. Alongside this there has been increasing international attention to the potential role that health services can play in helping to identify and support women who have experienced abuse and facilitate their referral to specialized services [3-5]. The majority of studies on intimate partner violence (IPV) describe pilot projects or small interventions, often run with time-limited donor funding. There is limited discussion about how these could be moved forward from small scale or pilot projects to nationally scaled interventions. Scaling up services for IPV is an important task in order to address the high prevalence and wide range of health consequences of IPV. Existing literature on scaling up complex interventions more generally points to several barriers, which are also relevant when scaling up integrated IPV services. They can include a lack of infrastructure and equipment; inadequate drugs and medical supplies; shortage and distribution of staff; negative staff attitudes; financial shortages; weak management, technical knowledge and inadequate supervision [6-8].

Despite limited debate on strategies for scale-up of IPV services, many countries are actively seeking to develop a health sector response to intimate partner violence (IPV) and sexual violence (SV), and are integrating violence focused services into different health service entry points, such as family planning, maternal health, and accident and emergency services [5,9-14]. Alongside these initiatives there has been a growing body of evaluation research exploring the potential role of the health sector in providing services to women who have experienced violence. The literature has focused largely on exploring provider and client attitudes towards health sector involvement, and evidence on the effectiveness of different forms of intervention activity. Much of the existing debate has focused upon whether women should be ‘screened’ for violence, and the extent to which such interventions impact on rates of case detection and women’s future risk of violence [14-19]. There has been much less debate about what may be the most feasible in different settings, and the relative merits of different entry points, and models of training and support, identification and referral. Enabling factors have included: partnerships between the health-care sector and other statutory bodies and NGOs; the importance of having a VAW policy; a multi-disciplinary response with medical, social and legal interventions; a joint surveillance system [20].

This article contributes to filling this gap by highlighting the constraints and enabling factors behind the scale up of the one stop crisis centre (OSCC) intervention to address violence against women - and particularly IPV - in Malaysia. We describe the results of a qualitative study of experiences with scale-up in two states: Penang and Kelantan.

## Malaysia OSCC response to gender based violence

Malaysia has been at the forefront of the development of One-Stop Crisis Centre (OSCC) - an integrated health sector model that aims to provide comprehensive care to women experiencing physical, emotional and sexual violence. The first OSCC was established in 1994 in the Accident and Emergency (A&E) Department of the General Hospital in Kuala Lumpur, in collaboration with women's NGOs. In 1996 the Ministry of Health directed all state hospitals to set up OSCCs for women survivors of violence, located within hospitals’ Accident and Emergency Departments [21]. Since then, this model has been replicated in several countries in the South East Asian region [6-8].

According to the OSCC guidelines developed by the Kuala Lumpur hospital, its main aim is to provide round-the-clock patient-centred services for abused women and children in one site, with the potential benefit of geographical proximity to all services, reduced or no delays for examination, ease of referral to specialized and non-health services [22]. The model of comprehensive service provision includes medical care, counselling, police aid and social support. Internal referral systems have been created to refer cases from OSCCs to other specialized services on-site; while an interagency network including police and social workers facilitate external referrals to social welfare and NGOs.

Key elements supporting the creation of the OSCC include a basic legal framework through the adoption of a national law on IPV in 1994, a strong NGO women’s movement, and many committed individuals, including in senior positions within the Ministry of Health (MOH) [23]. As part of the national scale up, hospitals were told to identify and set up a separate room to offer services to abused women, and to adopt the standard protocols for sexual abuse and child abuse developed in KL. This paper presents an analysis of providers experience with the national scale-up of the OSCC intervention in two contrasting States in Malaysia, located in the North East and the North West of the country, with the aim of drawing out the lessons learned for the future replication and scale up of health sector responses to gender based violence in other middle income settings.

## Methods

This was a qualitative study focusing on seven hospitals on the two Northern States, covering 2 tertiary hospitals and 5 secondary and district hospitals. Between January to April 2007, 74 semi-structured interviews were conducted with: 54 health providers (including nurses, medical officers, gynaecologists, medical social workers, counsellors and hospital managers) responsible for providing services to abused women; 8 policy makers at regional and ministerial levels; and 12 key informants (see Table 1). Very few counsellors were interviewed as they are primarily available at specialised hospitals, but also because counselling to women who experienced abuse is offered primarily by medical social workers.

**Table 1:** Details of in-depth interviews

**Staff profession**	**Number of respondents (male/female)**
Medical doctors (A&E)	9/14
Gynaecologists	2/4
Nurses	0/14
Medical social workers	2/3
Counsellors	0/2
Psychiatrists	2/2
Policy makers	1/7
Key informants	3/9
**Total**	**19/55**

Snowball sampling was used to identify health providers and key informants who were knowledgeable about the research topic and the researched model, with the assistance of the local partners. Respondents were selected according to their profession and their experience with violence issues (primarily violence against women) and their connection with OSCCs.

Semi-structured interview guides were developed for each respondent type as they offered core questions around their views about violence and their challenges when providing services to abused women. Respondents were asked about providers’ experiences in offering services, the challenges and opportunities they faced, and their views on the adaptation and scale-up of OSCCs. The structure of the interviews tried to encourage respondents to discuss matters they might have otherwise not revealed without prompting such as their beliefs towards IPV. It also ensured that issues relevant to the research were not completely overlooked and allowed comparison across sites and between States.

Prior to the fieldwork, the guides were tested before use, with the support of the local senior partners from the University Sains Malaysia (USM), and further refined and finalized. After piloting the instruments, the guides were modified accordingly and some questions simplified and refined also during the interview period. The majority of interviews were conducted in English. Malay interviews (primarily with nurses) were conducted by two local research assistants, who had been previously trained on the interviews-topic guides.

These interviews were complemented by a document analysis of hospital protocols, and site observations in each facility. In particular, a brief facility checklist was developed for facility observation and was used to collect information about the services offered at the selected facilities. For instance, it contained questions about hospital characteristics (e.g. number of beds, outpatients, services provided), about OSCC features (staff, services on site and on referral, observation of the room), about guidelines and protocols used, and about training offered. These data will be used for describing the visited settings in another article.

Digitally recorded interviews were saved onto a computer and subsequently transcribed. Malay transcripts were translated into English and their accuracy was checked by a local person and the field supervisor in Kelantan.

A framework analysis method was used to analyse the emerging themes. The transcripts of in-depth interviews were read repeatedly to familiarise with the text, to have a full picture of the data collected, and to begin to identify some main themes throughout such initial reading. Once finalised the cross-cutting thematic code framework, interviews were coded and managed by using NVIVO (N7), a qualitative software package (N7) used to help analyse and code narrative texts.

All through the analysis, the code framework was further revised to add new categories of codes, where new sub-codes and themes were identified. The coding system was further refined when we began to group together some linked themes and we came up with fewer broad overarching issues, linked to the main study objectives:

health providers’ attitudes and views (e.g. their views on IPV, on their role, on abused women, etc.). This contains a series of sub-themes that reflect providers’ characteristics as individuals;

challenges faced by the health providers when managing OSCC cases at health care delivery (e.g. lack of time, lack of training, etc.). This contains sub-themes about challenges met at individual level while delivering care, but also at organisational and health care delivery level (e.g. lack of human resources, lack of appropriate training, little collaboration, etc.);

challenges at policy-making level (as perceived by policy-makers).

Once coded, relevant information related to each main theme was combined and examined more closely.

Data were triangulated internally by interviewing different groups and through the use of a variety of data collection methods. The same issues were explored with different study groups (at health service and at policy level), in individual discussions and in different States. In addition, dissemination workshops helped verify that our own understanding of some of the issues were truthful to the providers’ views.

### Ethical issues

Ethical approval was granted by the Ethical Committees of the LSHTM, the World Health Organisation Scientific and Ethics Review Group (SERG), and the national Malaysian ethical review committee. Additional permissions to conduct interviews with health providers in the selected hospitals were also obtained by the State Health Departments in Penang and Kelantan.

Written informed consent was obtained from each participant in the study. Each informant was asked to sign an informed consent form and anonymity of all information was assured by asking them whether they agreed to be quoted in disseminating reports. A copy of the consent form was given to each respondent to keep. A Malay version was also available for respondents who would not be comfortable in English.

### Confidentiality

A coding system for all research sites within countries was put in place. In particular, confidentiality of records, tapes and transcripts was assured through numeric coding. Interviews were recorded, where consent was given to tape. Each interview was coded and no name appeared either on the informed consent or on the checklist. In addition, when the respondent agreed to be taped, a recording code was attached without the name of the interviewee, so that the respondent would not be identifiable by the transcribers. Transcripts had no signs of identification for participants. Care was taken in ensuring that any quotes used during the dissemination were anonymised to ensure that the individual respondents/participants could not be identified.

## Results

**Figure 1:**
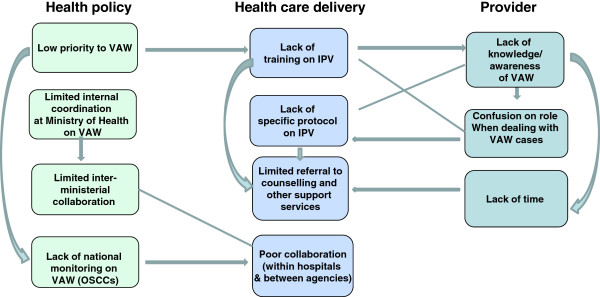
Flowchart on the knock-on effects of different challenges across levels.

The findings from our in-depth interviews show that the implementation of the OSCC model varies substantially and is influenced by a complexity of individual, systems/organisational and policy-level constraints. Our findings illustrate the plurality of factors affecting support for providers delivering front-line care in the scaled-up provision of IPV services in Malaysia, and the strong connections across individual, health care delivery and policy levels. Figure 1 shows a summary. The findings are structured around the three levels identified – policy, systems and provider – but highlight the interconnections at each stage.

### Policy level factors: political priorities and commitment

The OSCC creation has been guided by a policy directive from the MOH in 1996. Subsequently, specific training, guidelines and equipments have been developed to integrate OSCC services within all A&Es. Table 2 describes the policy guidance and key issues that characterises Malaysian OSCCs.

**Table 2:** Policy guidance and other OSCC key elements

**Policy support**	1996 directive of Ministry of Health to develop a specific directive to manage abuse cases in general.
	Aim: to have a place where all services could converge so that the woman would not need to go to different places.
	Types of GBV: OSCC should provide services to women and children for any type of violence they experienced.
	Main role of the hospital: to provide a place for the OSCC within the Emergency Department where victims could be referred to when they didn’t need emergency medical treatment.
**Available clinical guidelines**	MOH developed a protocol on the management of rape that was disseminated to all hospitals.
	Guidelines for the hospital management of child abuse and neglect were published by the Ministry of Health Malaysia in 2009. Clinical practice guidelines (management of child abuse and neglect) are also available.
	A flow chart for child abuse is also available in public hospitals. No specific protocol or procedures have been developed specifically for IPV cases.
	Specific guidelines on the management of GBV cases - coming through OSCCs - were developed by the Kuala Lumpur Hospital (HKL) in 1994, and to be later used in all hospitals around Malaysia. However, not many hospitals are using these specific guidelines, but would use instead specific clinical protocols for rape and child abuse contained in these guidelines.
	Effort is currently being made by HKL to develop Clinical Practice Guidelines (CPG) for OSCCs by the end of 2012 (Wong 2011).
	At the same time, in 2011, the Malaysian Department of Social Welfare developed a document to ensure multi-sectoral cooperation when handling cases of domestic violence (Siti Hawa Ali - direct communication).
**Training**	Besides a national training course offered annually by the hospital Kuala Lumpur, very few specific courses are available in other States (e.g. Kelantan, Kuantan, Pahang), which focus primarily on forensic evidence and medical intervention. With the exception of Kelantan’s main tertiary hospital, the training courses do not run annually, have a limited number of participants, are often offered by specialised hospitals, and only few providers can attend them.
**Services provided**	Based on a multi-sectoral approach, OSCC provides comprehensive services such as counselling, medical care, support services, police and collection of forensic evidence, legal aid and temporary shelter, all in one site. Counselling is often offered on site by medical social workers at specialised hospitals, and upon referral by women’s NGOs or social workers from the Department of Social Welfare. Internal referral systems are created to refer cases from OSCCs to other specialised services on-site; while an interagency network including police and social workers is crucial for external referrals.

Despite the existence of a Violence and Injury Prevention Unit at the Ministry of Health (MOH) since 2004, the interviews with policy-makers at national and regional levels seemed to suggest a limited commitment from the top level management towards a response to violence against women. What emerged from the findings is that IPV and in general, violence against women, seems to have a lower priority than other key health issues within MOH. Less visibility seemed to be attributed to IPV because - as some national policy-makers mentioned - there were other “*competing priorities*”, which got more attention than violence issues. Consequently, OSCC services and IPV programmes have received little attention and fewer resources in comparison to more mainstreamed clinical issues.

Several respondents used the lack of any national prevalence data on violence against women to justify the scarce priority attributed to IPV and MOH action at regional level.

"..I tend to pay more [attention] on diabetes, more trainings, more everything.... more for the cardiovascular diseases.… because these are the diseases that we can see, the numbers, how many are dying, how many are getting the complications. Domestic violence, I don’t have any figure. (Policy-maker, female respondent)"

This view of IPV being a “lower” priority in comparison to other medical issues had multiple impacts, including on the management of the services at the hospital level, in the organisation of national and regional training as well as in the distribution of funding.

Despite the 1996 policy directive on OSCCs, in both States, some policy-makers said that they received very limited guidance about OSCCs, and complained that there was no national monitoring of the policy implementation.

"..So we don’t have the instruction from the top.. then we cannot lay out what to implement. There is nothing top down… policies, programmes, activities and all that. They don’t really monitor what’s happening (Policy-maker, female respondent)"

Thus, at the policy level despite a legal framework in place, lack of leadership from the top meant that there was little commitment to monitoring the IPV situation, providing data on its prevalence, which in turn led to health providers giving it lower priority compared to other health issues which were clearly supported by data showing them to be a problem. In addition, guidance on how to establish or maintain OSCCs was very limited. However, the joint alliance of women’s NGOs and some medical doctors helped the issue to be placed on the policy agenda [23].

### Health systems and organisational issues

Health providers identified a range of issues that can be described as systems or organisational factors that they felt constrained their ability to respond well to women seeking their help. These were generally seen across hospital levels although were often exacerbated at the lower levels of care. The nature and extent of referral and collaboration between relevant departments and agencies was constrained at all levels, but especially at secondary level facilities were specialists were few. Supervision and monitoring appeared to be an issue at both levels of care as was a perceived lack of training on IPV. Human resources revealed itself in terms of staff shortages across hospital levels, but with secondary and district hospitals additionally constrained by staff having duties across different parts of the hospital, further limiting their time at the OSCC. Each of these issues is now discussed in more detail.

#### Supervision, monitoring and training

Several providers expressed confusion about the case management process, due to the lack of clarity in the standard operational procedures (SOPs) for OSCC cases, particularly for domestic violence. In particular, some staff at specialised and district hospitals were sometimes unsure how to proceed with IPV cases, what injury to document, in what detail, how and what questions to ask, where to refer women. Some felt that procedures and protocols for domestic violence were not as thorough as the ones for rape or child abuse, and the process was less tight.

Moreover, despite the availability of some annual national and regional courses, some health providers at both tertiary and secondary care levels complained about the lack of proper training on IPV, as the programme offered was mainly focusing on rape cases and on medico-legal procedures, with very little information about domestic violence cases and how to inform women on local support and legal assistance (though mentioned in the nationally issued OSCC guidelines [22]).

#### Collaboration and referral

A key element of the OSCC model is to provide health, legal and welfare services on site. Collaboration and referral were less problematic at tertiary hospitals, because of the availability of specialists on site. However, linkages with external agencies for social support were still an issue also at this level. From the providers’ accounts and the results of the facility observations, it is clear that instead of being a “one stop”, the process may at times be lengthy and fragmented, especially at district facilities, where there were no specialists on site and very little collaboration with external agencies. When asked about challenges, many professionals said that collaboration and referrals between district hospitals and the relevant agencies were not functioning properly. Despite the MOH guidelines stating the role of each agency [22], there seemed to be little clarity at the ground level across partners.

"…one stop means that all people come over here to treat the patient, from the social welfare, from the police, they come over here. But so far this is not happening at all, so far if they come over here, they [doctors] treat the physical problem, physical injury problem, and then the social problem, we refer to them, and then they say “ok, go and see them over there”, rather then they come over here to see the patient, or even if they want to come, they … [are] on leave, not enough staff, so they cannot come. So, finally they have to go out to see them again. So, actually, the purpose is good but functioning wise is not practical. (District hospital, male respondent)"

Both States’ providers described the challenges associated with trying to provide services in settings with extremely limited referral options:

"… The most difficult challenge is to call them [support agencies] to come here for the particular [OSCC] case after office hours.... Also no NGOs here, only police and social welfare… (District hospital, female respondent)"

This lack of referral options at times resulted in hospitals temporarily admitting women into hospital beds, either because they had concerns over the woman’s immediate safety, or because the counsellor from the local NGO was not available at night.

#### Human resources

Scarcity of staff was a constraint expressed by various providers in both States and across hospital level. Staff sharing was another challenge reported by various health care professionals, especially at lower level facilities, where nurses and doctors were also shared with other units. Designated staff in charge of OSCCs often have to fulfil other additional tasks, as part of their daily work, besides their OSCC duties. The same person who is in charge of the OSCC may also be in charge of the casualty ward.

"[..] we don’t have enough team members. For OSCC actually, we do have team members, but the team member is the same who is in charge of the ward… (District hospital, female respondent)"

Various providers cited time pressure as an important constraint to providing quality OSCC care. Lack of staff leads to time shortages that have an impact on the ability of medical officers to identify cases of abuse, with some saying that time limitations made it more difficult to detect the real problem behind the injury - especially when women did not disclose. It was also cited as a barrier to asking the proper questions to assess women’s emotional needs for further referral.

"…we don’t have enough time, because a lot of cases.. the outpatient cases after office hours also come here, so we don’t have enough time to go in the separate room, to take a long history , so what’s usually happens, we are not going to ask the reasons why she was battered and go in deep depth on that. We just ask about what time, place, what weapon or thing that was used to hit the woman, do the physical examination then ask whether they are willing [to have] some counselling or refer… (District hospital, male respondent)"

Data from the interviews suggest that while there are real time constraints, whether or not they result in limited care depends on the individual interest of health professionals on violence issues.

### Provider experiences

The range of issues facing health providers involved in delivering services to women experiencing violence is detailed elsewhere [paper still unpublished]. Here, we focus on issues that are key to enabling providers to support clients within the health system context – some findings (e.g. poor knowledge of IPV procedures) were similar across hospital levels but providers at the district level faced particular frustrations and constraints because of a lack of resources and external support.

First, ensuring providers have adequate knowledge to properly understand IPV and the procedures for responding to women who have been abused is important for shaping providers’ attitudes, their perception of their role and the way they therefore treat clients. Second, adequate resources and health systems support also influenced, or exacerbated, providers’ perceptions of their role.

Most providers seemed to be against domestic violence describing it as “unfair” and “illegal”. Only a few health care workers at district hospitals played down abuse referring to it as a “small thing”. Despite this, most providers did not think of IPV as a public health matter, but viewed it more as a family or social issue, leading to physical and emotional harm; some providers acknowledged that some IPV minor problems could become severe if not addressed.

Most health care workers showed considerable empathy towards women. Sensitive and sympathetic attitudes reported during the interviews ranged from trying to console women, to gain their trust during examination, to respect and protect their privacy and confidentiality, to listen to them, to reassure them and make them feel not judged. A local study has pointed out that although many of the health care providers have generally showed understanding of the issue and support the effort to improve the OSCC services, there is still some confusion regarding gender related issues linked to IPV and SV [24].

Nevertheless, we found that when providers follow the traditional role of treating and solving IPV as a “medical problem”, they tend to focus on the physical aspect of the injury, minimise the underlying cause of the problem and ignore emotional care for patients. This arose largely from a feeling of inadequacy. Providers frequently reported feeling under-trained and poorly supported to help women beyond merely treating their physical injuries. While the concern of some providers was about whether they were counselling women properly, others stated they felt lost or anxious when they had to deal with cases with minor injuries and few bruises, as their health training for A&E would focus more on severe forms of trauma.

"We will try to offer whatever we have… we are giving them some suggestions where they can find solutions. That’s the only thing we can do, more than that I don’t [know]… (Specialised hospital, female respondent)"

At district level particularly, it seemed that providers’ perceptions of their role seemed also to be influenced by the level of resources available in their settings.

"There’s very little we can do… we can only reassure them that there’s nothing wrong, medication, tell them we’re around here if they need anything. (District hospital, female respondent)"

Despite resource constraints, the OSCC setting within A&E was considered important with many providers stating that abused women used doctors and hospitals as their first entry point for a “help call”, even when they had minor injuries. Some doctors at tertiary facilities stated they played an important role as they were the first entry point for women, offering “*a portal*” to find solutions and “*options*” about available services.

".. I think it is the duty of the medical officer to inform women of their options because they will not go directly to the women crisis centre [..] (Specialised hospital, male respondent)."

## Discussion

What emerges strongly from these findings is the importance of adopting a health systems-approach when integrating IPV services. The policy context in which the services are implemented has to be considered as it provides the basis for how the health system responds in terms of its resources and organisational structure for delivery of services. Providers’ own experiences and perceptions are in turn influenced by the resources and support available which affects how client-centred they are able to make their care.

The findings illustrate that, by focusing on the linkages between these multi-level factors (providers, health care delivery, and health policy), a scaled-up response can be fully effective since it could positively impact on the continuity and quality of the IPV care offered.

Although much of the existing literature found that culture and personal attitudes of practitioners are the main barriers to addressing IPV [25-27], this article shows that the lack of uniformity in the OSCC implementation is not only influenced by the negative views of individuals, but also due to many other factors, related to the structure and organisation of the health system (e.g. poor supervision and support; lack of clear guidelines on how/where to refer women; staff time being squeezed because of requirements across level clinical areas), and external policy constraints (e.g. the lack of political urgency which means there are few data or targets which could help staff prioritise OSCC work). These elements are closely intertwined and interdependent, which is corroborated by studies from industrialised countries [28,29]. Much of the existing literature focuses on just one aspect of providing services to abused women, but fails to offer a holistic view of the whole system in which services are provided and the connections between the levels, which this article has highlighted as important - and a systems approach needs to be taken into account in the many VAW interventions now being developed.

In Malaysia, very little training is available to regional staff – usually a one-off short training session - and only a few providers at district levels ever attended any seminars. Prior to 2011, training for OSCCs’ services was left to the creativity of the state hospitals (tertiary specialised facilities) and Department of health. However, by the last quarter of 2011, the Ministry of Health requested that all state’s health departments run a training programme for OSCCs at least once a year. It also requested to form a state level committee to monitor activities related to health sector’s response to IPV and SV (Siti Hawa Ali, direct communication). Most states have responded positively to the training and at the end of last year the state of Kelantan has formed the state level committee for IPV and SV. This effort will be monitored closely by the Ministry of Health in order to ensure its sustainability.

Existing literature shows that training and guidelines can be effective in the short term and could lead to an increase in referrals to support services [30-34]. However, it is clear from the study that these elements alone are not enough to sustain long-term changes within health settings. The findings suggest that structural and organisational constraints are also partly dependent on health policy and management issues. For example, the limited budget committed to OSCCs impacted on the implementation of IPV services as well as the little knowledge among agencies about their respective roles.

Moreover, although the OSCC guidelines mention the roles of various specialists, interdepartmental and interagency collaboration is hindered by the lack of any clear common procedures for IPV cases, and by the low commitment to such cases among support services. Limited inter-agency collaboration is common for other issues that may require a multi-sectoral response, such as with HIV care [35].

Health service interventions addressing VAW in other settings have largely focused on micro-level factors of the intervention, such as staff attitudes and behaviours, their lack of knowledge about abuse [36], and the challenges of screening [15,37,38]. There is less documented experience of how to address the broader structural elements that may compromise the effectiveness of programmes in the mid and long term and that may limit the potential for replication. Comprehensive health interventions cannot be scaled up without additional resources, but also without a functioning health system [39].

The findings illustrate that the one-stop characteristics of OSCCs are not realistic at lower levels of hospital care, where it instead transforms into a ‘many stops’ refer out system. Although the principles behind OSCCs are the same across hospitals, their implementation has differed across facilities and levels of care, with associated constraints due to the diverse actors involved, the differing hospital structures and organisation, and the local context and resources. Despite being nationally implemented, the Malaysian model was a fairly inflexible package for OSCC services (primarily developed for tertiary facilities), which was constrained by local resources. Moreover, despite the focus on multi-agency network and convergence of all services in one setting, the results show that this may not be possible at all levels of hospital care, especially at district level. This suggests that there cannot be one single integrated model for all levels of hospital care (like the one piloted in Kuala Lumpur), but probably each level should have an adapted version of the approach, reflecting the local resources and the context.

It may be worth exploring whether different models of integrated services for abused women could be adopted through different levels of integration. In general, degrees of integration of IPV services should vary by levels of care and resources available. It could vary from comprehensive services offered in one site (facility-level integration) - primarily at tertiary and in some secondary settings - to more basic services (like medical care and basic counselling) with a strong referral system to higher-level hospitals and local support services (provider-level or systems-level integration, primarily at district levels). Standard Operational Procedures and processes of care should also consider the availability of local resources and thus be adapted accordingly. Moreover, the referral system within the various levels of health care should be strengthened, as well as linkages between hospitals and other support services.

**Figure 2:**
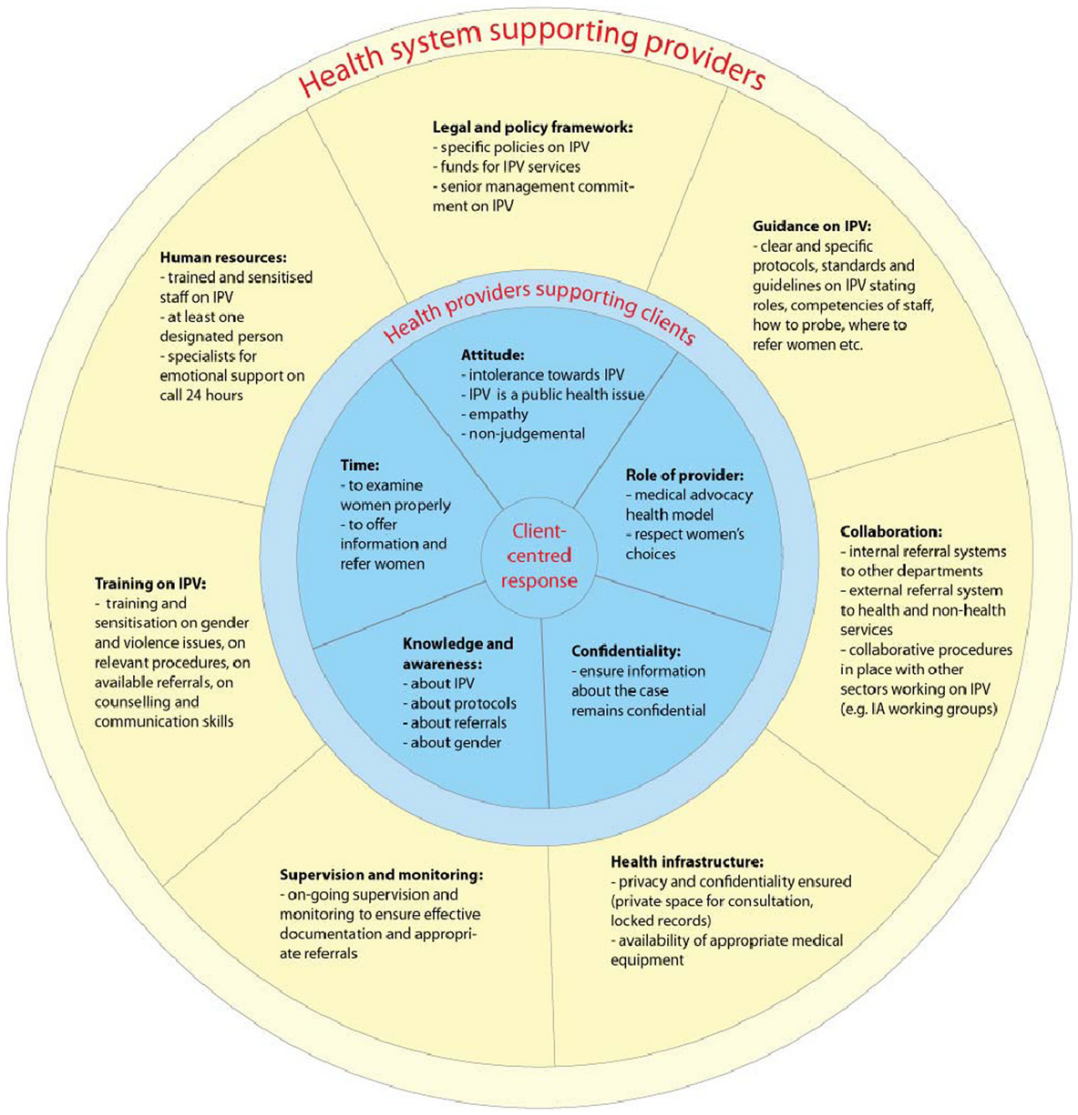
Wheel for supporting health systems responses to IPV.

Current debates still exist about the ways of addressing IPV and at what level a health response should be feasible, given the scarcity of resources. Based on our findings, Figure 2 illustrates what components need to be in place in order to support providers to offer a comprehensive and client-centred response when addressing abuse. It is based on the assumption that all elements – individual, organisational, contextual and structural – impact on IPV integrated care and should thus be taken into account. Integrating IPV into existing settings is not just a single-factor intervention, but encompasses a multi-level approach of various elements that require actions implemented by different actors at different levels and sectors. Some challenges may be dependent on organisational or structural issues, and if ignored, may make health responses fail.

### Limitations

One limitation of the study relates to the fact that by using qualitative interviews only, we gathered self-reported attitudes and views of health providers, but not their actual practices. Participant observation was not possible within the scope of the study. Therefore, we focus primarily on provider-related barriers and perceptions to the management of IPV, thus self-reported data could overestimate (or underestimate) some of the challenges in managing IPV cases. However, it is a valuable technique to further understanding of staff attitudes and their development.

Moreover, because of the limited sample size and the specificity of the findings, it may not be possible to generalise this study’s findings to other contexts in Malaysia, or to settings outside Malaysia. Nonetheless the findings can help understand what contextual factors may facilitate or hamper the provision of comprehensive IPV services, and therefore be useful to health practitioners and planners in other similar settings that may be in the process of scaling up IPV services. For instance, health systems constraints are quite similar across settings and thus may be more generalisable.

## Conclusions

When we discuss how the health sector can respond to GBV, we are always confronted with a complexity of challenges and barriers. The national implementation of OSCC provides a potentially important source of support for women experiencing violence. This article has identified the health systems factors necessary to support front-line health providers for successful scale-up of the current OSCC pilot intervention in Malaysia – lessons which can be applied elsewhere. Specifically these are: staff training to ensure sensitised have a clear understanding of what to do; changes to care structures to ensure designated OSCC staff have sufficient time to implement the procedures; privacy, that may or may not be linked to a specific OSCC room; and commitment and priority given to IPV at policy-level that will enable the production of data on violence and its inclusion in health indicators that will also give the issue visibility at the service delivery level.

The successful replication of the OSCC model in other similar settings requires that the model – and the system supporting it – needs to be flexible enough to allow adaptation of the service model to different types of facilities and levels of care, and to available resources and thus better support providers committed to delivering care to abused women. Even in more resource-constrained settings our findings show that staff who take the initiative can adapt to provide some level of OSCC services, such as referring women to local NGOs or community support groups, or training nurses to offer basic counselling – and if a health systems approach is taken to supporting such initiative the impact will be much greater.

## Competing interests

None identified. The author(s) declare that they have no competing interests.

## Authors’ contributions

All the authors read and approved the final manuscript. MC drafted the paper, collected and analyzed the data used in the article. CW contributed to the analysis of the main findings and to the final drafting of the paper. SHA and RS contributed to the data collection and its preliminary interpretation. SHM contributed to the drafting of the article and overviewed the development of the project.

## Pre-publication history

The pre-publication history for this paper can be accessed here:

http://www.biomedcentral.com/1471-2458/12/548/prepub
